# The model multiple: Representing cancer in sub-Saharan Africa

**DOI:** 10.1177/09526951241286733

**Published:** 2024-12-10

**Authors:** Jennifer Fraser, David Reubi, Thandeka Cochrane

**Affiliations:** King’s College London, UK

**Keywords:** Africa, cancer, epidemiology, models, multiplicity

## Abstract

Over the past half-century, modelling has come to play an increasingly important role in cancer research. These representational tools frame perceptions of malignant disease, guide public health responses, and help determine which interventions are necessary. But what makes a cancer model a model? What authority do they have? What stories do they tell? And how do they shape our understanding of disease and bodies? To shed light on these questions, this article explores the long history of cancer modelling in sub-Saharan Africa: a place where malignant disease has often been imagined as different, and where experimentation and improvisation in cancer research and treatment has been rife. Drawing on archival and ethnographic sources, we examine modelling strategies that health professionals have used to generate information about cancer in Africa from the mid 20th century to the present day. Focusing on three different case studies – anatomical models of Burkitt’s lymphoma patients, diagnostic models for Kaposi sarcoma, and statistical models of the African smoking and lung cancer epidemic – we meditate on the multiplicity of models and modelling by identifying the epistemic strands that hold these representations together, as well as what sets them apart. In addition to contributing to discussions of how cancer research has taken shape beyond the Anglo-American realm, our article helps expand and complicate our understandings of what a disease model is.

## Introduction

On 26 September 2022, a collection of international public health experts committed to strengthening health systems and enhancing global cancer control published a *Lancet Oncology* Commission on ‘Cancer in Sub-Saharan Africa’ (Ngwa *et al*., 2022). In this document, experts engage in an in-depth analysis of the state of cancer on the subcontinent and list key actions to enhance cancer research, reduce care inequities, and strengthen national cancer control programmes. A striking feature of this report is its ubiquitous use of models. To describe the profile and scale of Africa’s cancer burden, commissioners relied on GLOBOCAN – an influential set of estimates of the incidence of and mortality from malignant disease calculated by the International Agency for Research on Cancer (IARC) using a combination of empirical evidence and predictive modelling techniques (ibid.: e252, e254). The report also ‘highlight[ed] case studies and successful models that could be emulated, adapted, or improved across the region to reduce the growing cancer crises’ (ibid.: e254). In showcasing successful cancer prevention programmes from Africa and beyond, the report called forth numerous economic, training, and service delivery models as templates or exemplars to be scaled up across Africa in parallel with other national initiatives (ibid.: e265).

When we think about modelling in epidemiology, we often think about the mathematical and computerized simulations that shape how we imagine and seek to control epidemics. There are many examples, from the complex mathematical models that power the Institute of Health Metrics and Evaluation’s Global Burden of Disease estimates, to the computer simulations of the malaria epidemic run by the Gates-funded Malaria Atlas Project, to the modelling techniques undergirding the GLOBOCAN estimates mentioned above. COVID-19 has further entrenched the notion that epidemiological models are inherently mathematical and policy-focused. As Warwick Anderson (2021: 176–7) has shown, ‘statistical modelling’ and its language, from R values to ‘flattening the curve and epidemic waves’, has ‘pervaded public health policy’ and ‘public debate’ during the pandemic. Our tendency to equate epidemiological models with mathematical formulas and policy action also shapes how we think about modelling historically, as evidenced by the significant place given to the Ross-MacDonald model of mosquito-borne pathogen transmission in this Special Issue. However, mathematical, and statistical models are not the only form epidemiological models take. Indeed, far from being limited to statistics and policy, models take numerous forms and are used in varied ways to bring attention to issues, simplify complex phenomena, facilitate research, and effect change.

The heterogeneity of models is well trodden ground in the history and philosophy of science. As Mary Morgan has stated, ‘There are lots of different kinds of things that legitimately count as models in the sciences and the way these models look and function take drastically different forms’ (Morgan, 2012: xvi). In this article, we apply these insights and thinking to the world of epidemiology. Reconditioning Anne-Marie Mol’s (2002) classic title *The Body Multiple*, we draw attention to the varied ways in which epidemiologists and medical researchers have used models to represent, understand, and intervene on disease. Like Mol, we hope to show how things that on the surface appear singular, like models, are actually complex collections of values, materials, and practices, each saddled with their own ontologies and epistemologies. For us, models are material and conceptual analogues of physical or social worlds that come in all shapes and sizes: from tinker toy models that chemists use to visualize molecules, to mice that biomedical researchers employ as model organisms in experimentation, to mathematical formulas to model and manage the economy. As these examples suggest, models can be ‘more or less useful’ across a range of domains, from ‘applied and pure science’ to ‘public policy’ (Sismondo, 1999: 247). Some models are ‘thought-tools’ (Morgan, 2012: 10) that ‘create new knowledges and practices’ (Löwy, 2006). They are not just ‘representations’ of reality that are ‘schematic, miniaturised, [and] simplified’ but also ‘research objects’ that can be ‘manipulated and reasoned with’ (Morgan, 2012: 13). Other models are ‘more instrumental’ – ‘black boxes’ or technical apparatuses that are simply ‘feeding into other research’ (Sismondo, 1999: 256–7).

To convey the multiplicity of models and modelling in epidemiology, we explore some of the ways in which epidemiologists and medical researchers have used models to represent, understand, and intervene on cancer in sub-Saharan Africa. Both cancer and Africa represent compelling sites to articulate our argument. Cancer is a notoriously difficult disease to model. Its complex, multifactorial aetiologies, multiple expressions, and slow, non-linear progression generates ‘epistemic uncertainties’, making developing models for this condition highly challenging (Engelmann, 2021). Africa, imagined as a ‘living laboratory’ whose perceived cultural and environmental differences offered endless possibilities for medical experimentation, has long held an important place in cancer research (Cochrane and Reubi, 2023; Tilley, 2011). In the late colonial period, there was a surge of cancer studies across the continent. Working under the banner of geographical pathology, researchers hoped that the oncological exploration of Africa would yield important insights into cancer aetiology and help prevent the disease in imperial centres. More recently, during the height of the AIDS epidemic, Africa became an important research site in global oncology once more, especially for virus-related forms of malignancy like Kaposi sarcoma (Livingston, 2012; Mika, 2021). As we will show, the proliferation of oncological research efforts across Africa transformed the continent into an especially productive site for diverse forms of cancer modelling.

In this article, we focus on three specific efforts at modelling cancer in Africa, which we examine in turn and compare to give a sense of the different possible ways to understand, construct, and use models. While these are not the only ways that cancer has been modelled in sub-Saharan spaces, each of these examples were tremendously influential, reveal something different about how models operate, and raise important questions about what constitutes a model, what kinds of models there are, and what kind of information and impressions feed into their production. We start with the Lopez model, which sought to map the spread of the smoking and lung cancer epidemic across a succession of stages. Developed in the 1990s and highly influential across Africa over the last 30 years, it is more of a typical model made up of curves, numbers, and graphs. We then go back in time to look at a diagnostic model, a list of clinical symptoms developed in the 1960s that enabled doctors to identify the rise of Kaposi sarcoma and chart the later AIDS epidemic. Finally, we turn to a collection of plaster models used as a research tool by Denis Burkitt to help identify and map the geographical distribution of Burkitt’s lymphoma in the 1960s.

Focusing on these three case studies, we meditate on the plurality of forms that models can take by identifying the epistemic strands that hold these representations together, as well as what sets them apart. We also emphasize how these models and the ideas they project about cancer and Africa both ‘mirror’ and ‘shape’ the colonial and postcolonial ‘social, economic and political conditions of their development’ (Löwy, 2006: 344).

### The Lopez model and the making of the African smoking epidemic

The Lopez model was devised by Australian demographer Alan Lopez and his team at the World Health Organization’s (WHO) Tobacco or Health Programme in the early 1990s (Lopez, Collishaw, and Piha, 1994). The end of the 20th century had seen a rise in global epidemiological surveillance and modelling efforts fuelled by improvements in computer technology, talk of new global threats, and a desire to rationalize health policy (Anderson, 2021; Jones and Greene, 2013; Reubi, 2018). The Lopez model was a product of this era. It sat at the confluence of a few influential epidemiological projects in which Lopez and his team were involved, from Richard Peto’s articulation of new methods to estimate tobacco-attributable mortality to Chris Murray’s estimation of the global disease burden for the World Bank (WHO, 1990; World Bank, 1993). These projects, like the Lopez model itself, were efforts to produce reliable figures on smoking to enable policy-makers to take effective action against the tobacco epidemic. This seemed especially critical in Africa, where there was a paucity of data and the epidemic had begun to grow (WHO, 1997).

**Figure 1:**
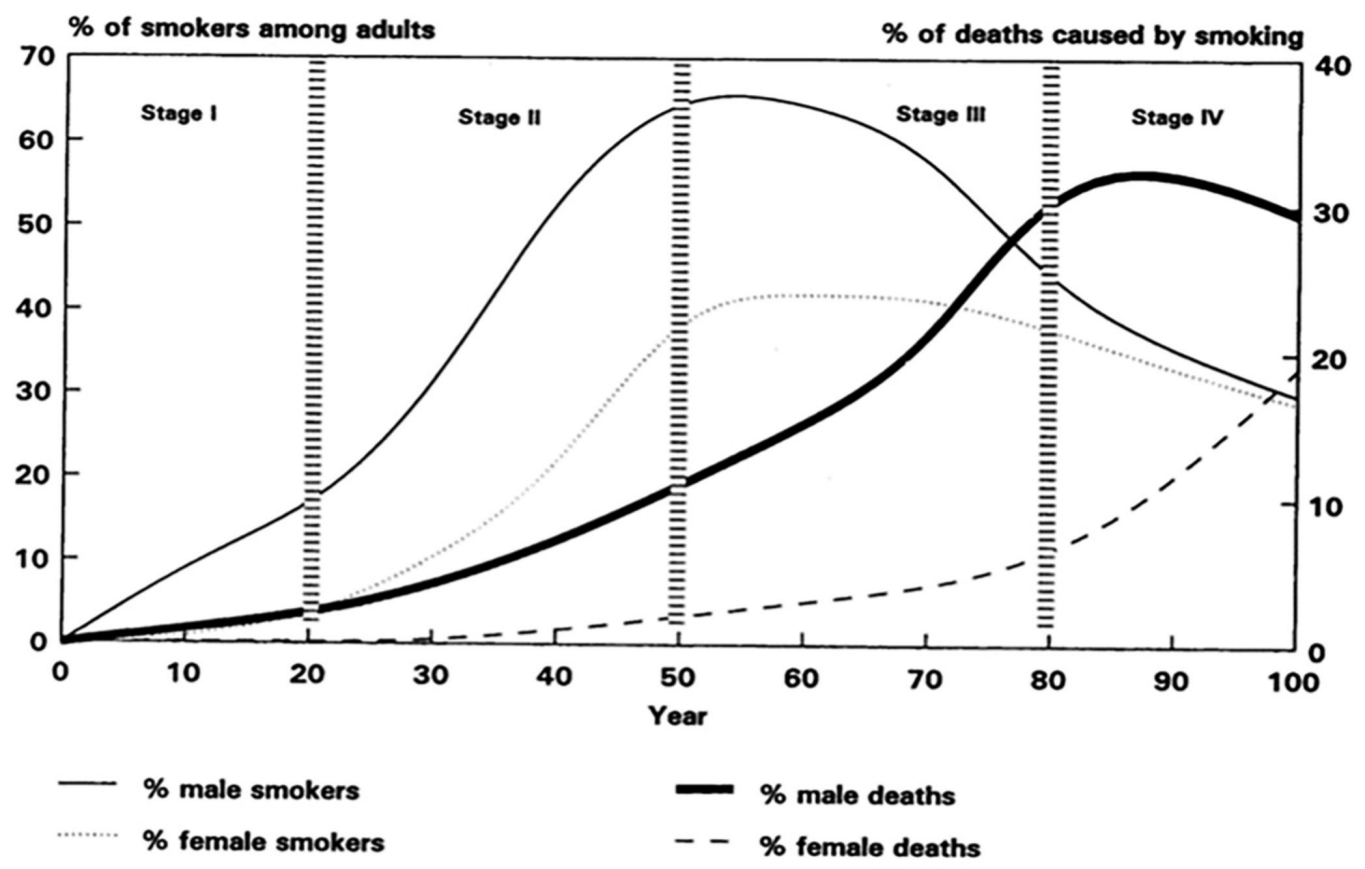
A model of the cigarette epidemic (Lopez, Collishaw, and Piha, 1994: 246).

The Lopez model is an account in words, numbers, and graphs of how the smoking epidemic unfolds in a population over time structured around four successive phases (see Figure 1). Stage I is the start of the epidemic: male prevalence rises up to 15%, ‘death and disease due to smoking are not yet evident’, and ‘tobacco control strategies remain underdeveloped’ (Lopez, Collishaw, and Piha, 1994: 244). Stage II sees the epidemic develop further: male prevalence continues to grow, peaking at 65%; female prevalence starts to increase to reach over 30%; smoking-related deaths among men start rising, mirroring the rise in smoking with a 20-year time lag due to the late onset of lung cancer; and tobacco control policies remain weak, with the risks of smoking still not widely understood. Stage III marks a turning point: male prevalence begins to decline to 40%, while female prevalence plateaus at 40% before decreasing; smoking-related mortality among men rises dramatically, accounting for 30% of all deaths; smoking-attributable mortality among women also starts to grow; at the same time, public attitudes to smoking change, with ‘knowledge about the health hazards of tobacco [now] widespread’ and tobacco control policies put in place (ibid.: 244–5). Stage IV is the tail end of the epidemic: smoking prevalence for both sexes continues to decline; smoking-related mortality among men begins to decrease, while it continues to rise among women, reaching 20%; and public attitudes to smoking harden and anti-smoking policies become more comprehensive.

Like other mathematical models, the Lopez model combines data sets and formulas. For the most part, Lopez and his team drew on data from ‘developed countries’ that had ‘experienced all four stages of the cigarette epidemic’ such as the US, Australia, and Finland (Lopez, Collishaw, and Piha, 1994: 242, 245). Specifically, the prevalence percentages used in the model come from two sources: data on prevalence ‘gathered through censuses and population surveys’ and ‘consumption figures from tobacco sales data’ (ibid.: 243). The origin of the mortality figures found in the Lopez model is more intricate. While acknowledging that ‘smoking causes mortality through a variety of diseases’, Lopez and his colleagues use ‘lung cancer death rates’ as the ‘index of total smoking-attributable mortality’ (ibid.). To determine how many lung cancer deaths can be attributed to smoking, they use an estimation method devised by Peto (Peto *et al*., 1992; WHO, 1990). As Lopez (1993: 92) discussed at the 1993 All-Africa Tobacco Control Conference in Harare, this method involved calculating ‘the relative risk of lung cancer death’ for smokers and, from there, the ‘aetiological fraction’ of all deaths from lung cancer due to smoking (ibid.: 92). This, he continued, is done by using ‘standard epidemiological formulas’ and working with a population for which there is good epidemiological data (ibid.: 87). Following Peto, Lopez and his team probably used US data and ‘adjusted [the figures] to compensate for different exposure history’ (WHO, 1990: 4). ‘The arithmetic of the tobacco epidemic’, they concluded, ‘is simple and stark. Cigarettes kill half of their lifelong users’ (Lopez, Collishaw, and Piha, 1994: 246).

Even though the Lopez model is a ‘descriptive model of [how] the cigarette epidemic [played out] in developed countries’, its primary policy message is for ‘developing countries’ that have not yet experienced the epidemic (Lopez, Collishaw, and Piha, 1994: 242, 245). This focus on ‘developing countries’ was not unusual back then. For a long time, health experts believed that smoking and lung cancer was only an issue for Western industrialized societies. But, from the late 1970s onwards, several publications with titles such as *Tobacco and the Third World: Tomorrow*’*s Epidemic?* (Muller, 1978) and ‘Smoking and Africa: The Coming Epidemic’ (Taha and Ball, 1980) warned against the dangers of growing cigarette consumption in developing countries, leading to international efforts to build tobacco control capacity across the South (Reubi and Berridge, 2016). Informed by this earlier work, Lopez and his team (Lopez, Collishaw, and Piha, 1994: 245) argued that while, for developed countries, the ‘model was historical in nature’ and, thus, of little value, it provided developing countries – especially in Africa – with an important epidemiological lesson and a chance to prevent a future public health catastrophe:

For developing countries in Stages I and II, it would be dangerous and erroneous to ignore tobacco as a public health problem because death rates due to smoking are low. If smoking prevalence is increasing, smoking-attributable mortality will inevitably increase [later].… [Developing countries] have the advantage of knowing the serious health consequences of smoking.… They can prevent history from repeating itself by taking strong public health measures to arrest the growth in tobacco consumption during Stage I or Stage II of the epidemic.… Many developing countries, primarily in sub-Saharan Africa, are currently in Stage I and, by undertaking preventive action now, can still prevent the cigarette epidemic from becoming a major cause of death in the future. (Lopez, Collishaw, and Piha, 1994: 245–7)

These ‘narratives of [future] catastrophe’ and calls for immediate action would often be reinforced by numerical evidence (Jones and Greene, 2013: 1208). In a 1995 article, for example, Lopez and his colleagues predicted that, by 2020, ‘the annual death toll from tobacco will have risen from about three million today to 10 million, with seven million of these deaths in developing countries’ (WHO, 1995: 297). Similarly, in his talk in Harare, Lopez outlined ‘the likely future annual [smoking-attributable] mortality among Africans’ (Lopez, 1993: 91). Noting that African tobacco consumption was, at ‘just under 400 cigarettes per adult per year’, ‘low but progressively rising’, he estimated that ‘25–30%’ of the 350 million under-19-year-olds then alive on the continent would ‘become regular smokers’ (ibid.: 90, 104). Anticipating that ‘between one third and one half’ of these future regular smokers would be killed by tobacco, he predicted that the ‘annual future mortality from smoking in Africa’ would eventually ‘reach 2 million’ (ibid.: 91, 104).

This talk of looming catastrophe and need for action found in the Lopez model assumes that the more a country is developed and modern, the further along the stages of the smoking epidemic it will have progressed (Reubi, 2016). This idea that a society’s socio-economic development is correlated with the maturity of its smoking epidemic is not unique to the Lopez model. Indeed, it can be found in many epidemiological writings on smoking in Africa (Reubi, 2020). Drawing on modernization theory and development economics, these experts tend to think that the modernization of the continent is leading to an increase in smoking and in smoking-related diseases like lung cancer. As a doctor working in francophone West Africa argued, lung cancer was a ‘pathology of development’ that had progressively emerged as the continent was modernizing and new ‘modes of life’ and ‘risk factors’ like smoking were taken up (Loubière, 1981: 34–5). The ways these experts have explained the relation between smoking and development varied. Some thought the relation was mostly economic, arguing that prosperity was an important predictor of cigarette consumption. Others emphasized the importance of cities, where many young Africans were migrating, swapping traditional village life for new Western forms of living in the process. Others still pointed to the role of women, predicting that female smoking would pick up as Africa modernized and women became more emancipated.

The Lopez model with its neat developmental stages, stark arithmetics, and talk of upcoming catastrophe has powerfully shaped global tobacco control imaginaries. The model has held sway among international experts in the field up to this day (Reubi, 2016). The article in which Lopez and his colleagues outlined the model has been cited more than 1600 times according to Google Scholar. In the 20-year anniversary issue of *Tobacco Control*, the Lopez model was identified as one of ‘the major achievements’ in the field and was the object of a study that confirmed the overall validity of this ‘landmark model’ in the light of new epidemiological data (Malone and Warner, 2012: 74–5; Thun *et al*., 2012). The model has also been successfully used by activists in policymaking, as with the adoption of the WHO’s *Framework Convention on Tobacco Control* (Reubi, 2018).

More importantly for us, the Lopez model has also been very influential in how the African smoking epidemic is imagined. To begin with, health experts working on tobacco control in Africa today commonly refer to Lopez’s model, borrowing many of its arguments and assumptions. For example, echoing Lopez, two researchers at the University of Cape Town recently noted that ‘while many African countries have low smoking prevalence [now], these countries will likely evolve to later stages of the epidemic with increased smoking prevalence’ in the future (Blecher and Ross, 2013: 6). To back their claim, the authors pointed to a couple of ‘critical warning signs’ (ibid.: 4). First, with the African population forecast to increase, there will be ‘growth in the number of smokers’ in the region even if prevalence remains constant (ibid.: 1). Second, Africa is, economically speaking, ‘one of the faster-growing regions in the world today’ and, ‘as the economy develops and incomes rise’, ‘so will smoking intensity’ (ibid.: 1, 4). All this, the authors claimed, shows that ‘Africa presents the greatest threat in terms of future growth in smoking’ and will be ‘the future epicentre of the tobacco epidemic’ if no action is taken (ibid.: 1, 3). Using an ‘updated [version of] the 20-year-old landmark Lopez model’, the authors even quantified the future rise in smoking, saying that from ‘77 million adult smokers’ today, ‘Africa will grow steadily’ and, in ‘the absence of policy intervention’, will reach ‘572 million by 2100’ (ibid.: 6, 8).

The Lopez model has also been influential among policymakers, funders, and activists working in the region. Indeed, they have used it to justify paying attention and allocating resources to tobacco control, even though smoking incidence and smoking-related diseases remain low in Africa (Reubi, 2020). The way the Gates Foundation (2015) justifies investing millions of dollars into tobacco control in the region is a good example:

We see great opportunities in … Africa. The tobacco epidemic in Africa is at a relatively early stage, so now is a critical time to invest in campaigns … that can prevent a large-scale epidemic. As incomes rise for a growing African population, tobacco use could double in the coming years if strong tobacco control measures are not implemented.

The African Union has used similar arguments to encourage member states to prioritize tobacco control, explaining that ‘Africa is still in the early stages of the tobacco epidemic’ and that African governments ‘must intervene now to prevent [future] tobacco-related death [and] disease’ (Zuma, 2013). African health activists, too, use these sorts of arguments when pleading for more tobacco control efforts in the region. As one East African activist explained to us in an interview,

Africa is in Stage I [of the Lopez model] with really low smoking prevalence and most smokers being men.… With economic development, also female empowerment and western lifestyles … this figure is going to change and if nothing is done now the epidemic will spread [and] we will have more non-communicable diseases.

Obviously, the Lopez model and the way it has helped frame smoking in Africa is not without problems. To start with, there are flaws with the ‘spatio-temporal logics’ of modernization that undergird the Lopez model and the way they rigidly order societies across the world along the same ‘epidemiological, geographical and development lines’ (Reubi, 2016: 188). Specifically, these logics assume that the trajectory of the smoking epidemic is identical all over the world, with the developing world inevitably following the path of Euro-America. This, of course, is often not the case, as Lopez and his collaborators themselves recently admitted in relation to female smoking in Africa, which did not take off as their model had predicted (Thun *et al*., 2012). Furthermore, by focusing mostly on prevalence, mortality, and policy, the Lopez model has little to say about the political economy of the tobacco epidemic and, especially, the role of the cigarette industry in the making of the epidemic. This has led to the erasure of crucial social and political realities, from the exploitation of small farmers and deforestation linked with the cultivation of tobacco to the aggressive marketing strategies and imaginaries of modernity used to sell cigarettes (Muller, 1978).

**Figure 2:**
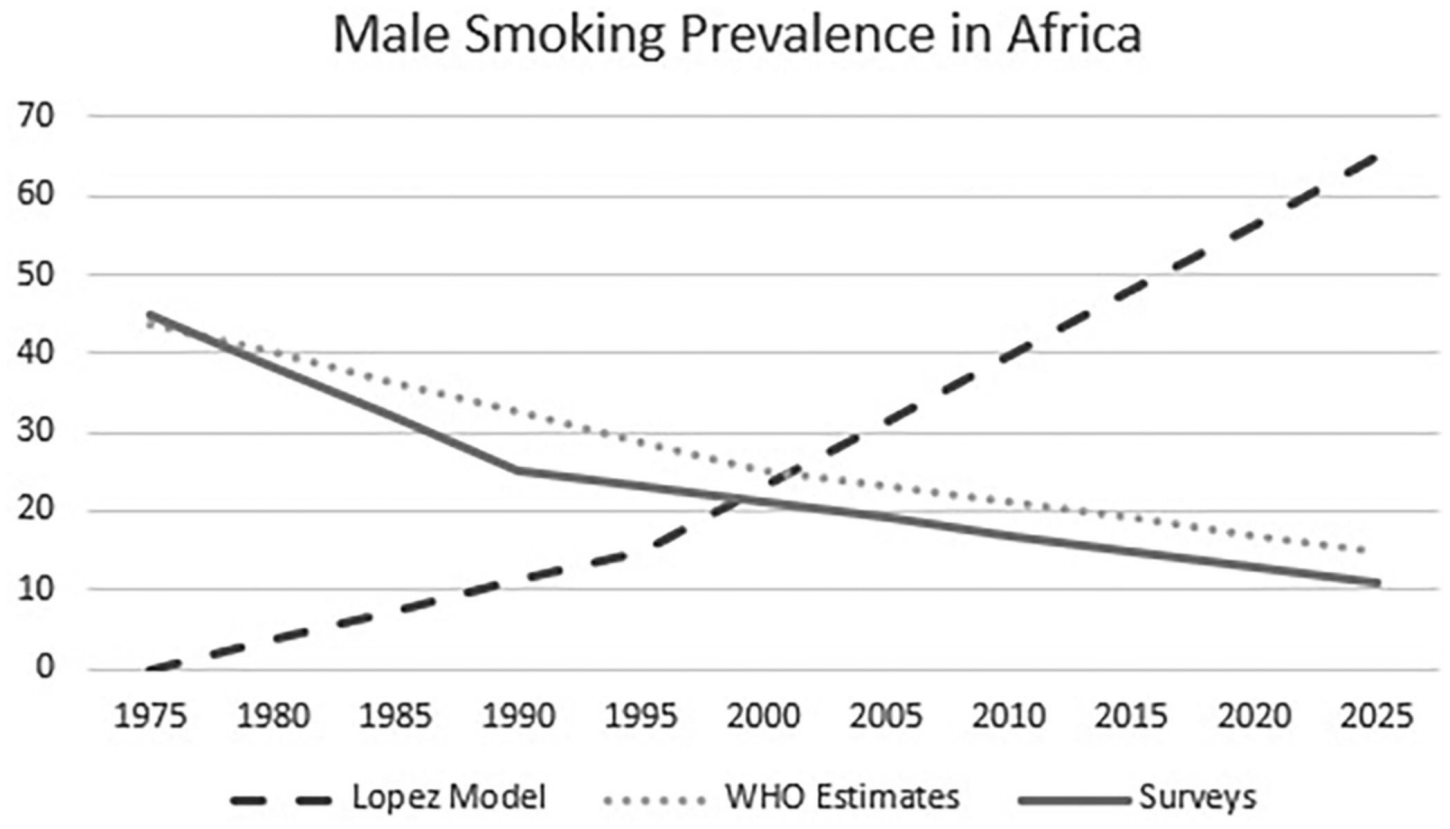
Smoking prevalence (%) among males in sub-Saharan Africa, 1975–2025, according to the Lopez model, WHO estimates, and existing survey data.

But what is most troubling with the Lopez model, given its promise to rationalize health priority-setting, is how it has led policymakers and funders to invest in tobacco control even though the story it tells about smoking in Africa does not seem to fit with the data on smoking collected on the continent (see Figure 2). Indeed, if we follow Lopez, Africa was in the first stage of the epidemic in the early 1990s, with male smoking prevalence at 15%, and would now, about 30 years later, be in the second stage, with male smoking prevalence at 65%. However, if anything, the epidemiological data available for Africa shows a decrease in smoking prevalence over this period (see Reubi, 2020). So, for example, the WHO’s estimates for the late 1970s indicated 40% for male smoking prevalence, while its estimates for the late 1990s and late 2010s showed 25% and 17%, respectively. Data from surveys suggests a similar trend. Surveys in the 1970s and 1980s measured male smoking prevalence between 40% and 50%, while surveys in the 1990s counted around 20–30% of men who smoked. More recent surveys, like the CDC’s Global Adult Tobacco Surveys, have measured male smoking prevalence rates from about 10% in Ghana to about 19% in Kenya (CDC, 2014). This downward trend is further confirmed by researchers from the Global Burden of Disease project, who have argued that male smoking prevalence decreased in most African countries between 1990 and 2015 (Reitsma *et al*., 2017). While this discrepancy between epidemiological data and the Lopez model is remarkable, what is perhaps more important here is how the Lopez model convinced policymakers and funders to invest in tobacco control even though smoking prevalence in Africa has been low and declining for the last 50 years. This is a potent if somewhat disturbing manifestation of the power of models.

### Malleable models: Kaposi sarcoma, AIDS and the co-constitutivity of clinical criteria

Diagnostic models refer to the codified tools and sequences that health professionals use to diagnose a disorder (Strauss, 1973). In addition to separating clinical entities and aligning them with specific causes and treatments, such models are indispensable for health data collection, the determination of treatment protocols, and public planning (Bowker and Star, 1999). They have also played an important role in how healthcare workers have conceptualized and responded to cancer in Africa. This is particularly true in the case of Kaposi sarcoma (KS) – a soft tissue tumour that usually presents itself in the form of painless, purple-hued lesions strewn across the body’s surface. The criteria for diagnosing this relatively rare, but highly visible, condition served as an important model. As this section will show, diagnostic models for KS not only helped researchers investigate, explain, and understand African cancer patterns, but they also shaped understandings of the distributions and origins of AIDS, one of the most recognizable and defining epidemics of the late 20th century.

Unlike the Lopez model, diagnostic models of KS did not take the form of growth curves, linear sequences, or developmental stages and were not attempts to simulate or foretell future epidemics. Rather, efforts to create an agreed-upon label for this condition were undertaken to assist clinicians with cognitive decision making and facilitate comparative research. However, as in the case of mathematical or statistical modelling, they propagated certain visions of the disease. As this section will argue, KS diagnostic criteria helped entrench ideas of African exceptionalism – ideas that would originally be applied to cancer and then, later, to AIDS. They also served as important empirical instruments, as KS case definitions have been indispensable for cancer and AIDS surveillance alike. In this way, KS diagnostic criteria function as their own ‘model multiple’. Serving as ‘malleable artifacts’ (Dacome, 2017), these clinical criteria were repurposed by different actors, at different times, to create and convey different meanings, collapsing the categories of chronic and communicable, endemic and virulent.

KS is a disease that has long-standing connections to Africa. The disease was purportedly first described by Hungarian physician Moritz Kaposi, who, in 1872, documented several cases of a pigmented sarcoma of the skin, typically found on a patients’ lower extremities (Shiels, 1986). At the time, the condition was thought to primarily impact elderly men of Jewish and Mediterranean descent. However, over the course of the 20th century it came to be increasingly associated with Africa. This was, in part, due to the efforts of Drs Comlan Alfred Quenum and Robert Camain, two researchers working at the Faculty of Medicine and the Institut Pasteur in Dakar, respectively (Camain, 1954; Quenum and Camain, 1958). While Camain was a French colonial doctor stationed at the Institut Pasteur in French Guiana before transferring to Senegal, Quenum was a pan-Africanist intellectual and doctor born in French Dahomey (Benin). He would later become the first African to serve as the regional director for Africa for the WHO (Hessoun, 2014).

In a 1958 survey, these men commented on the ‘fantastically high incidence’ and unusual presentation of KS among Africans living in some countries of Equatorial and Southern Africa – a finding that quickly captured the attention of researchers around the world (Rothman, 1962: 311). Their reports of the disease’s unique symptomology and high incidence coincided with a period when global interest in studying African cancer patterns was starting to take off. This interest was fuelled by geographic pathology, a precursor to modern-day cancer epidemiology, which saw researchers increasingly view group comparison as a crucial tool in the fight against non-communicable disease. In this context, Quenum and Camain’s results reignited interest and investigation into KS, a disease that had been known to researchers for the past 90 or so years.

Camain was a member of the International Union Against Cancer’s Sub-committee for Geographical Pathology in Africa. As a result, his and Quenum’s reflections on KS patterns quickly attracted the attention of numerous prominent geographic pathologists and spawned a series of spin-off studies across the continent (Rothman, 1963). These efforts culminated in a special conference in 1961. Held at Makerere College in Kampala, Uganda, this ‘Symposium on Kaposi Sarcoma’ was designed to draw attention to this malignant tumour that was ‘particularly frequent, or important, on the African continent’, and bring researchers together to discuss their findings (‘Introductory Statement’, 1963).

A central locus of discussion was whether, or in what ways, KS lesions in Africa differed from those observed in other regional settings. There was a lack of basic agreement about what KS was and how it exhibited itself in patients, with workshop attendee Jack Davies noting that some portrayals were ‘utterly at variance’ with others (Davies, 1963: 59). This seemed to be particularly true for African KS cases, as lesions identified in African countries seemed to possess key differences from those found in Europe and North America. In Western countries, KS was a relatively rare condition. However, in Africa, the disease appeared to be much more common, making up 9% (as opposed to 0.02–0.07%) of all observed malignant tumours. In sub-Saharan settings the age incidence of disease also appeared to be more variable. Whereas KS had traditionally been associated with later adulthood, in Africa it was ‘not uncommonly found in … children in the first decade of life’. Furthermore, while KS’s ‘extreme variable progression’ tended to be a well-recognized feature of the disease, symposia attendees reported that among African patients, KS tumours tended to be of the indolent variety, with a much steadier clinical course (Davies, 1963: 60–1). These differences caused many workshop participants to express concerns about KS case definitions. Most attendees agreed that there was a need to continue studying KS incidence in Africa. However, there was widespread concern that existing diagnostic criteria could not be relied upon to produce consistent and comparable statistics or generate new etiological knowledge.

This 1961 workshop had a marked effect on how KS research would be carried out. The notion that KS presented differently in African contexts, in terms of both its distribution and symptomatology, transformed the subcontinent into a hotspot for studies of the disease. By the early 1980s, a large body of literature had emerged from places like Uganda, Zaire, Burundi, Tanzania, Kenya, and Zambia (countries where the incidence of KS was the highest). Taking the form of registration forms, incidence tables, and dot distribution maps, these early models of the disease were researcher’s attempts to make sense of KS and generate novel knowledge about what was, at the time, a rather mysterious condition (Burkitt, 1973). By the 1980s these studies had become so voluminous that researchers decided to convene a ‘Second Kaposi Sarcoma Symposium’. Held in Kampala in January 1980, this workshop was an effort to bring together ‘eminent and interested workers in the field … to enable them to discuss recent advances [in KS research] and to make recommendations for future studies’ (Olweny, 1981: ix). Much like the earlier 1961 conference, KS clinical criteria played an important role in participant discussions. It was here that researchers first attempted to devise a clinical classification of the tumour that would better map onto its behaviour.

**Figure 3:**
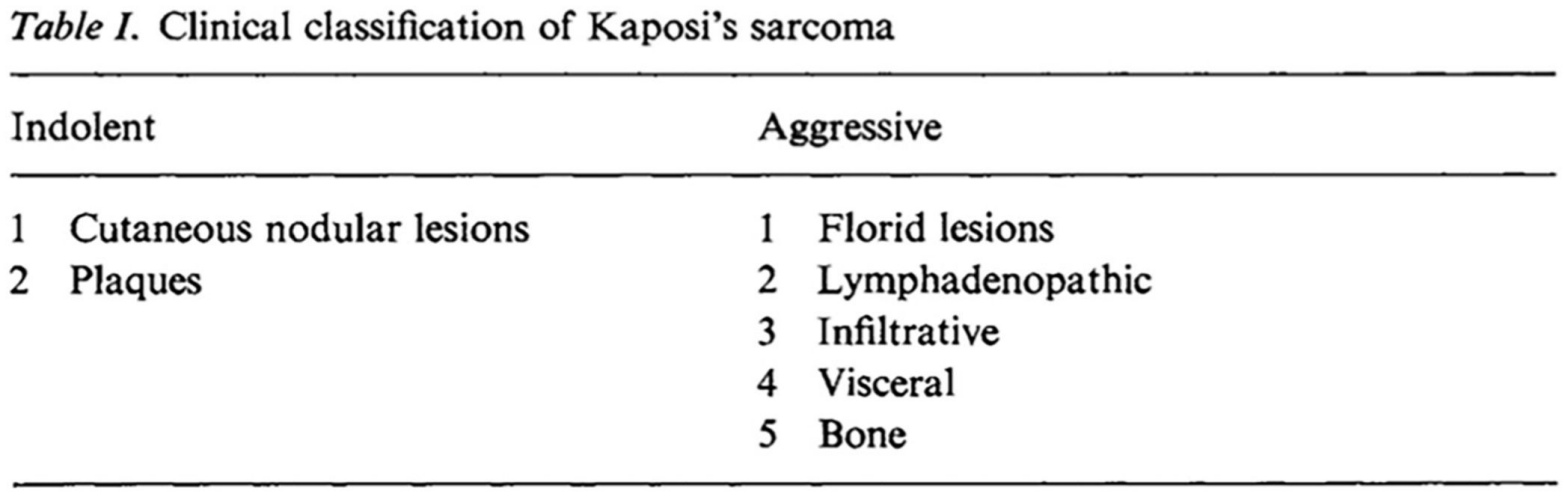
Sebastian Kyalwazi’s clinical classification of Kaposi’s sarcoma (Kyalwazi, 1981: 67).

Researcher’s experiences in Africa seemed to indicate that KS was not one homogenous disease, but a constellation of different conditions. Whereas some cases were confined to the skin of older adults – where they behaved as benign tumours – in children and young adults KS was primarily a disease of the lymph nodes. Here, its clinical course was more rapid, typically claiming the lives of patients in less than a year (Kyalwazi, 1981). To help health professionals distinguish between these subtypes, Sebastian Kyalwazi, the first African surgeon in East and Central Africa, used the Second KS Symposium as an opportunity to propose a new diagnostic framework (Figure 3). Drawing from his clinical experience, he suggested that all cases of KS could be divided into two categories: indolent or aggressive varieties. Whereas indolent lesions were slow-growing and usually did not result in death unless they involved a vital structure, the aggressive forms were highly malignant, often killing their victims within 8–10 months (Kyalwazi, 1981: 67).

Kyalwazi’s diagnostic model was well received by workshop attendees, as many participants believed that this division would improve diagnostic and prognostic accuracy. The bipartite structuring of KS also helped fuel the hypothesis that certain forms of the disease were associated with a viral infection. This was a key takeaway from the 1980 symposium. Building on a long tradition of tropical and colonial medicine, which often viewed the subcontinent as a cradle of transmissible disease, participants believed that studying the more ‘aggressive’ forms of KS would not only save lives but also serve as a ‘useful model for a study of the role of viruses in tumour etiology’ more generally (Symington, 1981). Kyalwazi’s framework would soon become even more prominent in the medical world. His separation of KS lesions into ‘indolent’ and ‘aggressive’ subtypes would form the basis of ‘endemic KS’ and ‘epidemic KS’, two diagnostic categories that emerged during the AIDS epidemic – a global public health crisis that took place just one year after the Second KS Symposium had drawn to a close.

Often appearing on the bodies of AIDS patients, KS quickly became an important marker of this largely unseen condition. Indeed, KS lesions served as an empirical and metaphorical reservoir for describing and interpreting the AIDS epidemic, acting as an important external signifier of this highly interiorized syndrome (Preda, 2005). However, few studies have emphasized just how central a role KS classificatory schemes played in AIDS diagnostics.

KS was an AIDS-defining illness. When the WHO first classified AIDS in 1985, it was a disease characterized by the presence of one or more indicator conditions. The WHO developed a catalogue of opportunistic infections that were afforded a numbered score based on their likelihood of being associated with the disease. When several conditions were present, reaching or exceeding a score of 12, a diagnosis of AIDS was established (Engelmann, 2018: 52). As Lukas Engelmann has shown, whereas pneumopathy had a score of 2 and herpes had a score of 4, generalized KS was scored 12 (ibid.). This meant that the presence of KS alone (no matter the subtype) was enough to constitute an AIDS diagnosis – even when there was no other evidence of disease.

During the mid 1980s, WHO officials created a modified version of this numbered system for use in Africa. Established at a WHO workshop on AIDS in Bangui, Central African Republic, this schematic was created to improve the accuracy of AIDS diagnostics in areas that did not have access to the sophisticated laboratory equipment required for HIV testing. While numerous conditions, such as weight loss, diarrhoea, or prolonged fever, were singled out as potential indicators of the disease, the presence of KS was seen as enough for clinicians to confidently make an AIDS diagnosis (De Cock *et al*., 1991).

Just as cancer research helped form the basis of AIDS diagnostic models, AIDS, a disease that would itself become a model for global health responses, also served as important scaffolding for cancer research (Brandt, 2013). Framing the disease as a sign and symptom of a developing outbreak provided cancer researchers with new sources of financial and institutional support. Throughout the 1980s and 1990s medical professionals who had spent their careers looking at African KS incidence began rebranding their studies as HIV/AIDS research to gain access to funding. Even large-scale cancer research bodies, like the International Agency for Research on Cancer (IARC), emphasized KS’s connection to AIDS to develop strategic partnerships, strengthen existing KS surveys, and acquire funding to keep floundering African cancer registries alive. As one researcher working at IARC in 1983 noted, cancer research in Central Africa had the potential to ‘hold the key’ for many important AIDS-related puzzles, including the tendency for KS lesions to appear on the bodies of relatively young, otherwise healthy, men, as well as the fact that so many of France and Belgium’s early AIDS patients appeared to have resided, at one point in their lives, in Central Africa.^1^ Capitalizing on this relationship also helped fund numerous KS studies and cancer registration efforts in Zimbabwe and Rwanda, showing how diagnostic models not only help doctors diagnose patients in the clinic, but can also impel change at the public policy and health systems planning level (Ngendahayo *et al*., 1989).^2^

While these tactics helped keep cancer epidemiology in Africa going, they also strengthened the relationship between KS and AIDS. For instance, African KS rates have been used by researchers to model HIV transmission and pinpoint the date when the first cases of AIDS were thought to occur in Africa (Zeleza, 1997: 40). The construction of a symbiotic relationship between these two diseases has also had a marked impact on how cancer data in sub-Saharan spaces is collected. In African cancer registration, comorbidity data is usually not recorded. The one exception is the case of HIV infection, as the upsurge of interest in the relationship between cancer and AIDS in the 1980s and 1990s resulted in the systematized recording of HIV status in many African cancer registry abstraction forms, making it appear as though KS is a by-product of HIV, instead of a stand-alone disease.

Correlating KS diagnostic models with those of AIDS has had several significant consequences. The intermingling of these frameworks has served to muster resources and mobilize ‘knowledge, deliberation, judgement and action to help stave off catastrophe’ (Anderson, 2021: 167). However, much like the Lopez model, these classificatory frameworks are deeply embedded in multiple and porous webs of meanings and relations. While these modes of classifying conditions may streamline diagnosis, they also collapse complex histories, pathologies, and sociopolitical realities. For instance, the WHO’s decision to make KS a key criterion of AIDS in Africa was met with some pushback. Since Africa had a long history of endemic KS, some practitioners believed that conflating these two conditions was leading to inaccurate diagnoses and health data collection. They argued that because some areas had a high background incidence of the disease, KS cases should be considered to indicate AIDS only in the presence of a positive HIV test result (De Cock *et al*., 1991: 1188; Gilks, 1991). Even though KS case definitions were originally created by African researchers who were interested in their research potential and, to some extent, therapeutic value, their integration into AIDS diagnostic models also helped cement AIDS as a uniquely African condition – a view intensified by theories of animal-human transition and the associated view that HIV was born out of a simian precursor found in Central and West African apes. Diagnostic categories of KS also mapped onto late 20th-century ideas of ‘African AIDS’ – the notion that AIDS in African and Caribbean countries had a distinctive clinical and epidemiological expression and could be classified as a more ‘endemic’ form of the disease, as opposed to the epidemic type usually observed in North America (Engelmann, 2018: 152–7; Kearns, 2017: 8).

The way that both KS and AIDS have been clinically defined has had important implications for how these diseases in Africa would be conceptualized. As we have seen, KS classificatory systems not only served as a model for AIDS diagnostics, but the AIDS epidemic also guided how KS data would be collected and used across Africa. In this way, these diseases modelled each other. Like Russian nesting dolls, these clinical case definitions fit into and expand one another, while never exactly replicating what came before.

To ‘model’ is, in a sense, to show the construction or appearance of something. Whether it be with clay, mathematical formulas, or computer software, the verb implies pliability – or the presence of a malleable substance that allows for the possibility of multiple forms. While clinical case definitions might not be the most obvious example of a model, looking at how KS has been represented over time helps us understand how viruses and cancers have been managed and measured. It also illustrates the multifarious nature of models, and how they are not one singular entity, but, rather, a patchwork of partially coherent and coordinated enactments (Mol, 2002).

### Wax models: Cancer moulages and the quest for aetiologies

**Figure 4:**
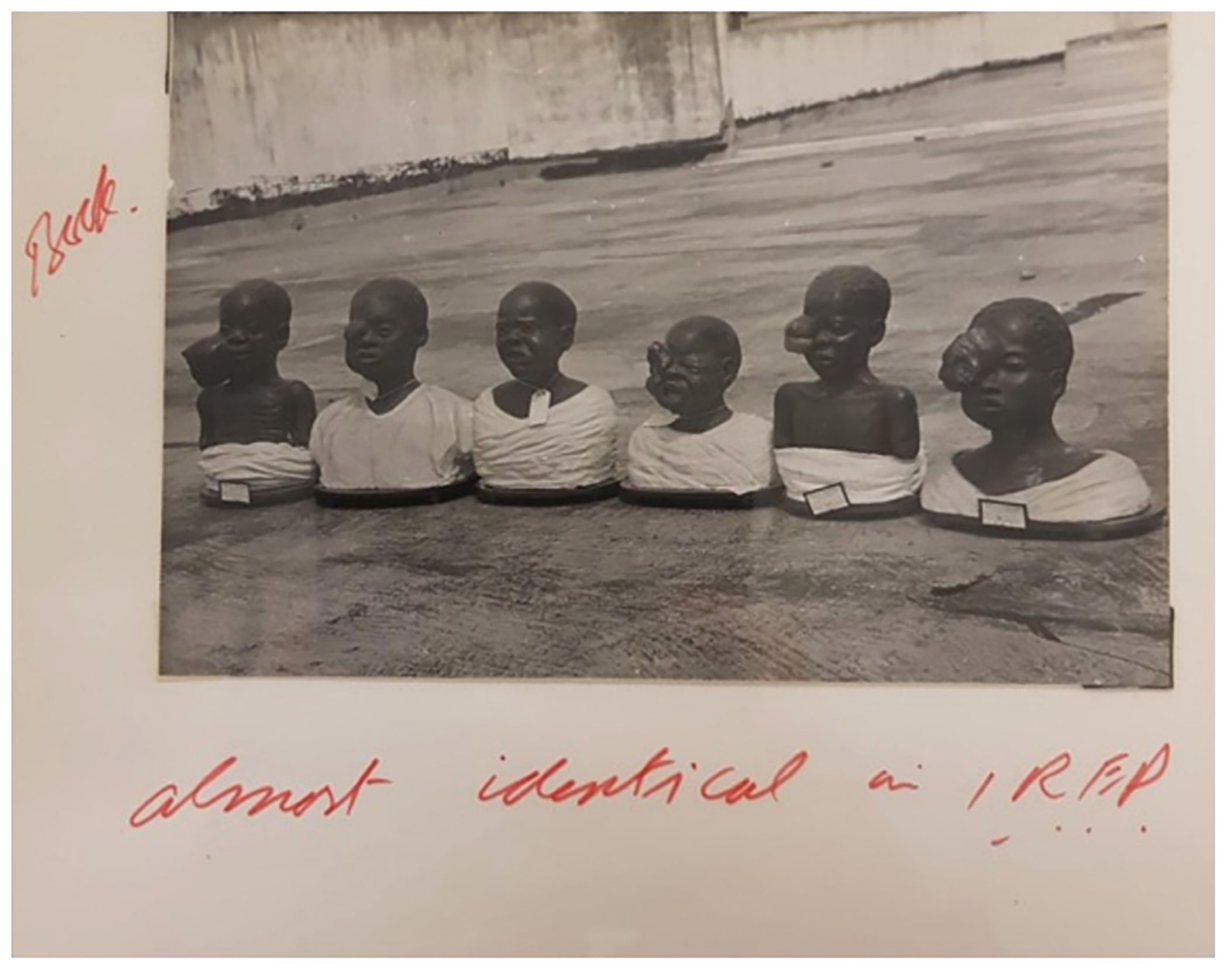
Plaster busts of young children with Burkitt’s lymphoma (WTI/DPB/B/7/4b ‘Album, maps and diagrams relating to cancers in Africa,’ Denis Parsons Burkitt Collection, Wellcome Archive, London, UK).

We turn now to a form of model that further troubles our notion of what constitutes a medical model: medical wax models. Here, we examine six plaster busts of young children suffering from Burkitt’s lymphoma (see Figure 4) - waxen plaster-cast models, and photographs of them, that were mobilized in an attempt, much like with KS, to establish a link between cancer and viruses.

These plaster models were the property of Manuel Prates, an eccentric doctor working at the central hospital in Lourenço Marques (present-day Maputo).^3^ Prates was a fervent collector of wax models, housing a vast number of wax impressions of diseases in ‘my museum’, as he called it, a vast pathological museum he curated in Lourenço Marques.^4^ Although owned by Prates, it seems the models were made by an unnamed museum worker, who had passed away by the time the models were photographed.^5^ The photograph was taken in 1961 by Denis Burkitt, a colonial medical officer in Uganda, highly regarded surgeon, and aspiring cancer researcher. In the early 1960s, Burkitt was trying to uncover the aetiology of a tumour that occurred primarily in young African children – later called Burkitt’s lymphoma. Burkitt famously reached to geographical pathology and an extensive and elaborate process of disease mapping to try to establish the cancer’s aetiology (see Reubi and Cochrane, forthcoming). He also, however, utilized photographs of a handful of plaster models of children disfigured by the disease. Burkitt came across Prates’s models while on a long ‘tumour safari’, travelling across East Africa to uncover the potential climatic and geographical causes of the jaw cancer (Reubi and Cochrane, forthcoming). When Burkitt and his friends arrived in Lourenço Marques in search of traces of the tumour, Prates enthusiastically showed his visitors his museum, including the plaster models.

Burkitt was deeply impressed by Prates’s models. An ardent photographer, he took pictures of these ‘lovely plaster models’ and ‘featured [them] prominently in [his] scientific publications and lectures describing the epidemiology of this tumour in Africa’.^6^ Burkitt believed that the pictures of the models ‘add[ed] real spice [to his] lectures’ and scientific publications.^7^

Interestingly, Prates refers to his plaster models as ‘moulages’. Moulages constituted a particular form of disease modelling that rose to great prominence in the 19th century. They are ‘pictures of disease in wax’ (Schnalke, 2004: 207). Moulages are a form of wax modelling derived by making a cast of the diseased body part of a person. Typically, this is achieved by placing a thin layer of cloth over the visibly affected part or limb of a patient and then layering plaster onto this (ibid.: 210). This plaster cast is then removed, and molten wax is poured into its ‘negative’ (Fend, 2022: 27–9). Once the wax version of the moulage is created, it is frequently mounted on a board and displayed as a ‘fragmented … malfunctioning’ part of the human body (ibid.: 45). Some moulages, particularly those of the face, require subsequent modelling work to be done afterwards, often in the presence of the patient, to depict those parts of the face that could not be plaster cast – such as the inside of mouths or open eyes as seen in Prates’s models (Schnalke, 2004: 211).

**Figure 5:**
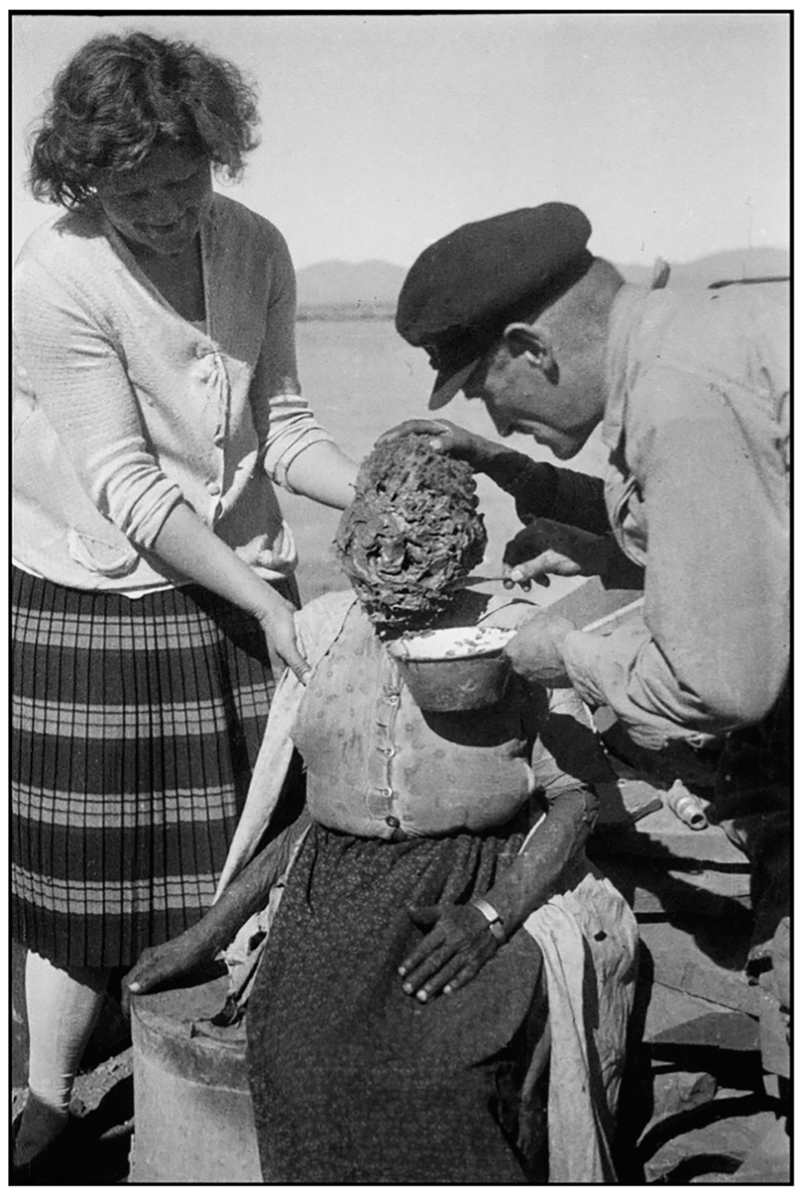
Licthenecker making a cast in Namibia (Wolfgang Wiggers Personal Archive, https://www.flickr.com/photos/15693951@N00/).

The process of plaster casting the faces of Africans was not new in the colonial history of the continent, particularly for ethnological purposes. In the 1930s, for example, Hans Lichtenecker, a German ‘raciologist’ (*Rassenforscher*) and Nazi sympathizer during the Third Reich, travelled around German South-West Africa (present-day Namibia) to make casts of the faces of its indigenous inhabitants in order to create an ‘archive of vanishing races’ (see Figure 5; ‘Encounters’, 2023; Hoffmann, 2015). The process of making plaster casts was a ‘suffocating and often terrifying procedure’, as is attested by descriptions from Lichtenecker’s journals (Hoffmann, 2015: 91). These accounts ‘give insight into the immediate violence of the project’, with one Indigenous Namibian man recounting how he ‘could not breathe.… [My] ears were sore, sore, sore.… I sweated wet, wet, wet, from sweat’ (cited in ibid.: 91, 96). The archives do not tell us how Prates’s models were made nor how much say the children had in giving their image over to be modelled. We can, however, infer that the process was likely to have been highly uncomfortable for the children subjected to it, not least because the casting of diseases, which capture broken, pustulated, wounded skin, is far more painful than normal plaster casting (Fend, 2022: 35).

However the moulages were obtained by Prates, they played an important part in Burkitt’s epidemiological work. In the 19th and early 20th century, moulages and wax models of disease were considered an important component of medical research, providing both exemplary pedagogic tools, but also objects to promote the understanding of disease (Maerker, 2013). Plaster models were key in helping physicians understand the ‘characteristic features’ of a disease so that the physician could experience an ‘identifying moment’ in which the ‘disease becomes a visible entity perceived as a clinical picture’ (Fend, 2022: 31–2). In this sense, plaster models, like other models of disease, produced a key tool for the process of seeing disease – knowing it and being able to act upon it. The plaster models of the African lymphoma that Burkitt photographed played a similar role. They captured ‘nature in the very act’, allowing the moulder to ‘fix, on solid matter, a fleeting and transient but characteristic moment’ (ibid.: 38). It was in this capacity to capture a ‘characteristic moment’ that the models were so crucial to Burkitt’s research to discover the aetiology of the African lymphoma. The clear distortions of children’s faces by multiple tumours made the lymphoma easily recognizable to the naked eye – marking this particular cancer as a highly visible one that did not require specialist tools of any sort to be ‘seen’.

Burkitt used the aesthetic nature of the tumour extensively in the process of collecting data on it. He believed that the distribution of this tumour indicated an environmental (or possibly viral) cause to aetiology, and so he hoped to gather data points on it from across the continent, which he then pinned onto a map (see Reubi and Cochrane, forthcoming). His first foray into collecting these data points was through a postal survey, containing information on the tumour, a questionnaire, and some photographs of afflicted children, that he sent to African clinics and health centres. Later, on his safari, Burkitt carried photographs of the tumour with him across Africa to show to medical professionals to confirm whether they had seen the tumour or not. However, once he had the photos of Prates’s models, the archives suggest that it was these images that he utilized in talks and speeches across the world, rather than the far more graphic pictures of children. Burkitt’s research eventually led to the identification of Burkitt’s lymphoma as the first cancer to have a proven viral aetiology, making the models part of a highly significant oncological discovery on the global stage.

The wax models that Prates made, and Burkitt photographed, might seem very distant from the mathematical and abstracted models discussed above. The models, however, throw into light some of the questions around what can be understood by the act of modelling disease. As Anna Maerker has argued, when thinking about anatomical wax models, we need to ask ‘what kind of representation we are dealing with in terms of materiality, mode of production and the narratives that surround them’ (cited in Fend, 2022: 36). These questions are fitting for all forms of medical models: mathematical, abstracted, as well as waxen.

The wax models and their photographs enacted important facets of models in general. They acted as tools for research and simplifications of a complex disease (to the reduction of a child’s bust) for more understanding and knowledge of the disease to be produced, as well as for the generation of more data on the disease. In this, Burkitt mobilized the ‘truth value’ of moulages and medical models, which were historically considered to have a very high degree of ‘verisimilitude’ and were trusted as ‘authoritative representations of the human body’ making them excellent scientific objects (Fend, 2022: 35, 51; Maerker, 2013: 532). Like with other forms of models, the representational authority that the moulages were seen to carry played a pivotal role in their use-value as research tools.

But as in the case of other models, such as the Lopez and KS models, this process of simplification also obfuscated the underlying colonial contexts and power relations that led to their production. As outlined above, the process of casting a moulage is long, arduous, complicated, and painful. And it was often undertaken within a matrix of hugely unequal power relations in which scientists subjected colonized bodies to their research. Throughout the continent’s history, it and its inhabitants were seen as a ‘living laboratory’ within which African bodies could be used for research without much concern for consent, and often through coercive measures (Tilley, 2011). Unlike with many other even more abstract models, the visceral nature of the plaster models means that their ability to hide the colonial contexts and power relations they are embedded in is limited. These limits, however, draw our attention to the ability of all models to obscure what lies behind them – the people that inform them, the power relations they are built on – many far more successfully than the plaster models of Burkitt’s lymphoma.

By using photographs of moulages rather than of children, Burkitt was able to utilize their high degree of ‘truth value’ as representations of the disease he was studying without bringing the viewer, his audiences, into too intimate contact with children’s suffering. As Fend (2022) has argued, photographs and medical models were configured as ‘indexical media’ for the display of disease. In fact, photographs long played an important accompanying role to that of the wax model – already in the 19th century, photographic anthologies of the wax models held in medical museums across Europe were a key means of distributing knowledge about disease (Pierce, 2020). Burkitt used his photographs in much the same vein in his research on the African lymphoma. His research relied heavily on doctors across Africa reporting whether they had encountered the tumour or not. The photographs of the moulages allowed Burkitt to present doctors with an image of some of the lymphoma’s most visually striking symptoms before asking them whether they had indeed *seen* the tumour or not. Moreover, unlike the ‘bulkier and more fragile’ moulages, the photographs were an ‘immutable mobile’ that could travel easily across vast distances (Hopwood, 1999).

The photographs of the models also allowed a particular degree of abstraction – a key element of what models do. In rendering the tumour of the children in wax, the models, and the photographs of the models, afford the viewer a particular distance from the disease while simultaneously evidencing the ‘pains somebody suffered’ (Fend, 2022: 52). In this, the models constitute ‘troubling objects’ that allow us an uncomfortable proximity to the distortion of the afflicted child’s body, while nonetheless permitting us a degree of distance (ibid.: 41). Although the models show the children with their eyes open, their mouths crying out in pain, we are not made to look directly into the eyes of suffering patients, as we would in photographs, nor are we turned into morbid voyeurs.

Despite offering distance through abstraction, the photographs of the models also rendered a particular form of value as ‘shocking objects’ (Schnalke, 2004: 209), or, as Burkitt phrased it, added ‘spice’ to his lectures – bringing a degree of texture to the tumour that was still palatable. Put differently, the photographs were ‘troubling objects’, which afforded the viewer a particular distance from the disease while simultaneously evidencing pain and suffering (Fend, 2022: 41). The photographs’ shock factor played an important part in garnering support for Burkitt’s research. By showing the distressing nature of his tumour, Burkitt was able to draw enormous degrees of attention, and thus financial support, for his research (Reubi and Cochrane, forthcoming). Models of all kinds often act to generate a sense of ‘crisis’ and a need for financial and research investment (Anderson, 2021). This is perhaps most clearly articulated in the Lopez model. However, this is not confined to predictive models of epidemics. Wax models mobilized in ways like Burkitt used them can evoke a sense of shock, and through this a form of crisis in the shape of horror that portrays bodily urgency and mobilizes financial and research investments. Ultimately, Prates’s wax models had both a ‘performative capacity’ and an ‘epistemic weight’: they were objects of knowledge that could be used to draw attention to and think about disease dynamics and patterns (Engelmann, 2021: 2)

## Conclusion

In this article, we have sought to elucidate the multiple forms in which medical models can appear through presenting a variety of attempts to visualize and make cancer legible in Africa. Cancer is a disease that resists modelling – its ambiguous nature, both infectious and non-infectious, its elusive aetiologies, and its unclear progressions make the disease difficult to grasp hold of. Nonetheless, as with all illnesses, models have been mobilized to create greater medical clarity. More conventional models, such as the Lopez model, have been applied to cancer in Africa, as have diagnostic models, such as the KS model, which articulates tensions between modelling and producing correlation. Even wax models have been mobilized in attempts to understand the disease, map its patterns, and pinpoint its aetiologies.

While the models outlined in this article all reflect different modalities of modelling, they share many of the commonalities of models in general. They all seek to simplify phenomena, explain patterns or correlations, draw attention to a particular issue, act as workbenches of epidemiological reasoning, marshal financial resources and research investment, and impel action. Through the three models, we have seen how processes of simplification and stabilization produce obfuscations and erasures. In the Lopez model, the political economy of tobacco is nowhere to be seen, nor is an awareness of Africa’s existence in a concurrent temporality with Euro-America. In the KS diagnostic model, we see how notions of KS and AIDS as separate and distinct conditions becomes lost, and in the Burkitt’s lymphoma models, we see how colonial contexts and unequal power relations are (partly) obscured. Equally, the three models show the different forms of epidemiological reasoning that models both mobilize and produce: from predictive modelling to warn against epidemics in the Lopez model, to clinical models of AIDS and KS to help see and manage a disease, to the instrumental models of Burkitt’s lymphoma, which sought to generate data points and undergird theories about carcinogenesis.

In her 2012 *The World in the Model*, Mary Morgan argued that while models may not be easy to categorize, to understand them, ‘we should pay attention to what models are used for and how they are used’ (Morgan, 2012: xv). In this way, she impels us to consider not only how models encapsulate the world, but the active role they play in worldmaking, and how they comprehend and reshape the spaces we inhabit. Through their various lenses and agendas, all the models explored in this article highlight the different ways in which ‘modelling involves social processes and practices that construct its inevitably selective readings of and gazes on the world’ (Leach and Scoones, 2013: 10). As we have seen in our case studies, ideas about Africa and its relationship to disease had a profound impact on the creation, interpretation, and logics of cancer models. At the same time, these models all enact different imaginaries about the continent. Whether it be ideas of backwardness, infectiousness, or inherent difference, these examples call forth certain tropes about Africa – by-products of the social and political worlds these modellers were born in that would be reflected in their work. Through their multimodality, these models call attention to the ‘manyfoldedness’ of models, and how these systems draw together a diversity of elements and rely upon many forms of coordination (Mol, 2002: 84).

## Notes

1 P. J. Fischinger, Letter to Lorenzo Tomatis, 3 November 1983, International Agency for Research on Cancer Archives, Lyon, France, G3/6: Coordination with the National Institute of Health, Bethesda, USA, Jacket 28–32, June July 1982 to December 1984.

2 M. T. Bassett, Interview by D. Reubi and T. Cochrane, 23 January 2023.

3 D. P. Burkitt, ‘Diary of the Long Safari’, 1961, Wellcome Archives, WTI/DPB/B/4, p. 38.

4 M. Prates, Letter to Harold Stewart, 1957, National Library of Medicine, Bethesda, USA, Stewart Papers, Box 29, Folder 4.

5 Ibid.

6 Ibid.

7 Ibid.
